# Pre-clinical evaluation of mRNA-lipid nanoparticles’ potency and toxicity: current practices and future directions

**DOI:** 10.1007/s44164-025-00096-5

**Published:** 2025-11-24

**Authors:** Chloé Muzard, Johanne Seguin, Jonathan Bonnefoy, Nahla Salkini, Vincent Serra, Khair Alhareth, Katia Lemdani, Nathalie Mignet

**Affiliations:** 1 Université Paris Cité, CNRS, INSERM, Unité des Technologies Chimiques et Biologiques pour la Santé (UTCBS), Paris, 75006 France; 2 Neovacs SA, Suresnes, France

**Keywords:** MRNA, Lipid nanoparticles (LNPs), Potency, Toxicity, In vitro assay, Novel alternative models

## Abstract

Over the last few years, the success of COVID-19 mRNA vaccines has resulted in the emergence of RNA lipid nanoparticles (LNPs) with promising prospects for the prevention and treatment of various diseases. The context of the SARS-CoV-2 pandemic has led to the rapid development of vaccines with abbreviated non-clinical programs. However, there are currently no official guidelines defining the required standards for global marketing of mRNA based therapeutic products. Nevertheless, to guarantee a well-controlled product, it is essential to characterize both the drug substance and the final product in terms of their structure, composition, formulation, physico-chemical features, potency, and safety. This lack of guidance has resulted in a wide variety of heterogeneous in vitro tests being used to assess the potency and cytotoxicity of RNA-LNP. This review discusses the commonly used in vitro assays, primarily 2D monolayer assays, employed to evaluate the biological properties of RNA-LNP. We then explore novel alternative methods to bridge the gap between in vitro and in vivo results. We summarize (i) co-culture models, (ii) multilayer 3D assays and (iii) in vivo replacement models, exploring their potential applications in assessing the potency and safety of RNA-LNPs. Finally, we discuss the use of in silico and machine learning as models for optimizing and predicting the biological behavior of RNA-LNPs.

**Graphical Abstract:**

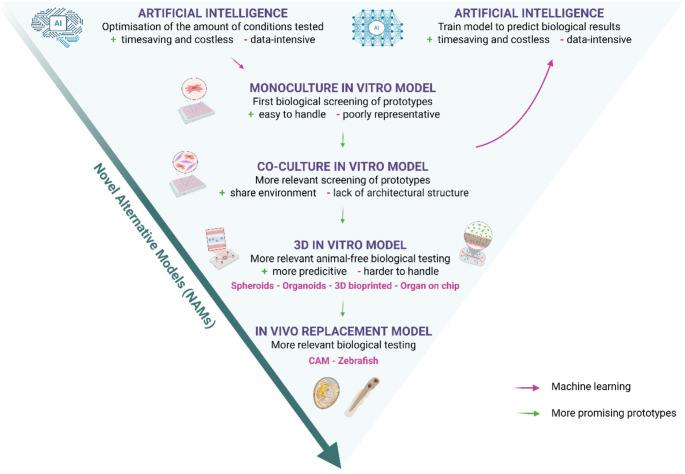

## Introduction

Given the success of messenger RNA (mRNA) vaccines developed in the context of the COVID-19 pandemic, RNA-based technologies have raised promising prospects for the prevention and treatment of various diseases [1]. Indeed, RNA encoding a wide variety of viral antigens; tumor associated antigens, cytokines, monoclonal antibodies and gene editing therapies are currently under preclinical and clinical development [2–5].

The efficacy of RNA therapy depends on the ability of the delivery system to bring nucleic acid to the targeted cells, which is essential to induce biological effects. In fact, the use of a delivery system is fundamental for a range of reasons, including protection of the payload against degradation, facilitation of membrane crossing for cellular uptake and tissue targeting. For decades, lipid-based delivery systems have been well established to encapsulate nucleic acids, with the use of the first lipoplexes and liposomes in the 90’s [6, 7]. The selection of novel cationic lipids, unprotonated at pH 7, and novel lipidic compositions has led to the description of lipid nanoparticles (LNPs) [7]. These nanoparticles have firmly established their position as the main delivery platform for therapeutic RNAs enabling higher encapsulation efficiency and improved efficacy [8–10]. Alternative non-viral vectors such as polymers, peptides and exosomes have been proposed, nonetheless LNPs remain the most used in clinical applications [11–14]. 

Comprehensive characterization of a pharmaceutical product is essential to guarantee therapeutic reliability and to comply with demanding regulatory standards, thus achieving a well-controlled product. Characterization of the final drug product (fDP) is essential to obtain knowledge about the structure, performance, and safety of the pharmaceutical product. In RNA vaccine development, characterizations are needed on both the drug substance (DS) (corresponding to the RNA), and the fDP (corresponding to the final formulated RNA). Physico-chemical characterizations as well as potency assay are required for each release of vaccine batches. The potency assay is a critical quality attribute that demonstrates the functional integrity of the antigens and supports the monitoring of batch-to-batch consistency and stability [15]. 

Today, industries are faced with various regulatory organizations with different requirements, according to the region of the world in which they wish to market their products [16]. The regulatory and reference documents regarding mRNA-LNPs development are limited and inconsistent between regulatory agencies. The United States Pharmacopeia (USP) and the European agencies have proposed draft chapters on Analytical Procedures for assessing the analysis of quality of mRNA and mRNA-LNP to support their development. Typically, physico-chemical quality attributes such as size, polydispersity, encapsulation efficiency, or mRNA integrity are studied during the development process [17]. 

The in vitro potency of an RNA vaccine, often referred to as a functionality assay, is assessed using a quantitative and validated method. The characterization of the correct protein size and identity expressed from the mRNA can be achieved by a cell-based or cell-free assay [17–20]. Moreover, the potency assay must respect some specifications, especially regarding the dose used. Therefore, to have predictive results it is necessary to adapt the potency assay according to the targeted application. Currently, there is no consensus on how best potency assays can be performed.

In addition to potency assay, toxicity and inflammatory responses should be assessed; as the innate immune system is highly sensitive to RNA molecules and LNPs components [21]. In this context, nonclinical toxicity studies in animal models are built to predict immune indicators, including reactogenicity, immune response, and toxicities. In addition, biodistribution and persistence studies are needed to determine whether RNAs or LNPs components are released into non-targeted tissues, and their duration of persistence [22–24]. Moreover, further studies are needed concerning the “novel” excipients, such as ionizable lipids, to support their safe use and assess their potential genotoxicity and systemic toxicity [25]. Animal studies are still required by regulatory authorities for the development of nanomedicine therapies but could potentially be replaced by accelerated transitions toward animal-free regulatory testing in the near future [26]. For this reason, it is of utmost importance to develop novel alternative methods (NAMs), allowing an optimal potency and toxicity evaluation of RNA-LNPs.

In this review, we will first describe in vitro potency and cytotoxicity assays, typically used for the development of RNA-lipid nanoparticles, and their limitations. We will then outline co-culture 2D models and innovative strategies based on 3D models such as organoids and organs-on-a-chip, as well as computational methods and their potential use for the study of potency and toxicity of RNA-LNPs. These models could bridge the gap between in vitro assays and animal study results, allowing for better predictivity and transposability to human safety and efficacy.

## Current practices: two-dimensional monolayer cultures for mRNA-LNPs assessment

The evaluation of RNA-lipid nanoparticles functionality and cytotoxicity are commonly assessed on in vitro models using immortalized cell lines or primary human cells. The choice of the appropriate cell type depends on several parameters, including the targeted organ, the route of administration, and the ease of cell manipulation. Immortalized cell lines are derived from sources with chromosomal abnormalities or mutations, such as tumors, and are advantageous for research due to their constant growth and reproducibility to proliferate indefinitely [27]. A various number of cell lines are available and differ by the phenotype (e.g. epithelial, endothelial, fibroblast), the organism (e.g. mice, monkey, human) and the derivate tissues (e.g. uterus, kidney, liver). Despite several advantages of immortalized cells, a major limitation to their use is that these cells will never be encountered in an in vivo organism, which may lead to a lack of transposability of findings with in vivo experiments.

Primary human cells are directly isolated from tissues, including blood and bone marrow, retaining the key characteristics of the source tissue, as well as inter-individual variability. This provides a model that more closely resembles human physiology, while generating more predictive data [28]. However, primary cells are harder to handle than immortalized cells, which explains why they are often used in a second intention to establish translational relevance especially when the therapeutic application targets specific immune subsets [29]. 

### In vitro assessment of RNA-LNPs functionality

The functionality of mRNA-LNPs is currently being studied using in vitro-based assays that determine protein expression in cells. A non-exhaustive list of studies describing (i) the assessment of protein expression after transfection and (ii) the trafficking of mRNA-LNPs reported in the literature are summarized in Table 1. A variety of immortalized cell lines, specifically human epithelial cells (e.g. HEK293, HeLa, HepG2…) are used [30–34]. HEK293 are widely used in RNA transfection assays exhibiting high level of protein expression making them ideal for RNA delivery studies [35]. Other cell types including muscle cells (e.g. C2C12, HSkMC) and immune cells (e.g. DC2.4, BMDCs, RAW264.7) are used in the context of mRNA vaccine studies. They are generally less transfected than epithelial cell types [36, 37]. These discrepancies may be associated to variable IFN-I signaling between epithelial and immune cells [38, 39]. Dendritic cells (DCs) internalize mRNA-LNPs through toll-like receptors (TLRs) and produce large amounts of IFNα, then mature, and stop proliferating due to the anti-proliferative effect of ISGs (interferon-stimulated genes). In addition, the secreted IFNα into the environment induce paracrine effect on the neighbor DCs instructing them to eliminate the foreign substance (i.e. the transfected mRNA) [38–42]. 

The transfection efficacy is mainly evaluated using well-known methods such as flow cytometry, fluorescence microscopy, or luciferase reporter assays [36, 43]. Overall, the various reported studies are consistent with the fact that transfection level is dependent on the lipid, the RNA sequence, and the cell type. For example, *Lo Presti et al.* highlighted the cell specificity in optimizing transfection efficiency by testing nine helper lipid-modified LNPs on several cell lines representative of the main liver, lung and spleen population [36]. *Shi et al.* compared the uptake and expression levels of mRNA-RBD encapsulated either in LNP, cationic nanoemulsion (CNE), or cationic liposome (lipo) in C2C12, DC2.4 and RAW267.4 cells. Interestingly, they observed different behaviors of the nanoparticles; while LNPs were mainly internalized by myocytes leading to humoral response after immunization, CNE and lipo were highly expressed in dendritic cells and induced cellular-preferred immunity in vivo [44]. 

Otherwise, cell-free translation (CFT) assays are being developed for rapid assessment of mRNA functionality without confounding their functionality by the quality of the LNP used to deliver the mRNA into the cells. The mRNA is directly delivered to the ribosomal machinery derived from several systems such bacteria, insects or mammalian cells. For example, *Stiving et al.* developed a CFT assay, using wheat germ extract, coupled to liquid chromatography-tandem mass spectrometry (MS) enabling the characterization and quantification of antigen proteins from a multivalent mRNA drug in a dose-dependent manner. However, the functionality of the mRNA formulated in LNPs (fDP) was performed in cell-based assay to provide a more representative output of its in vivo functionality [45]. 

To gain a better understanding on the RNA pathways, research groups are focusing on RNA-LNPs trafficking, including the uptake by the cells and the endosomal disruption [37, 46–49]. Indeed, LNPs are internalized by endocytosis into endosomes. As the endosomes mature, their pH becomes more acidic, inducing the destabilization of the LNPs, endosome bursting, and therefore the release of mRNA into the cytoplasm for translation by ribosomes. The timing and quantity of payload release from the endosome are crucial for efficient translation of mRNA. Various methods are thus developed to study endosomal escape [37]. Some studies have been carried out to analyze the intracellular distribution of LNPs using confocal microscopy, allowing subcellular localization [50]. For instance, fluorescent proteins have been used to label critical organelles (early, late, and recycling endosomes) of DC2.4 cells to investigate the influence of helper lipids on intracellular trafficking of mRNA [51]. Using the same method, a research group compared the subcellular trafficking of two types of LNPs on myoblasts [37]. Some works highlighted the utilization of galectin-based imaging assays as a marker of endosomal damage induced by the LNPs. *Munton et al.* used a fluorescent Galectin-9 reporter to assess the endosomal escape events induced by different mRNA-LNPs in various cell lines [52]. Recently, *Omo-Lamai and team.* demonstrated that galectin recruitment is strongly correlated with endosomal damages and LNP-induced inflammation, highlighting the need for fine selection of LNP according to the application [53]. 

Super-resolution optical imaging methods such as stimulated emission depletion (STED) microscopy and single-molecule localization techniques (SMLM, PALM, STORM) are developed to overcome the limitations of light sheet fluorescence imaging. These approaches are particularly powerful for tracking the intracellular trafficking of mRNA-LNPs, visualizing endosomal escape in real time, and correlating nanoparticle localization with specific endosomal markers at high resolution scale (20–60 nm), below the optical diffraction limit (250 nm) [54, 55]. For instance, *Paramasivam et al. *used SMLM to visualize mRNA within the endosomes and cytoplasm and resolved LNPs in sub endosomal compartments [56]. 

In addition, a complementary method has been developed to monitor the dissociation of mRNA from LNPs using Fluorescence Energy Transfer (FRET) [48, 57]. This method’s key benefit is that it allows quantitative molecular analysis (down to 10 nm), making it the go-to choice for mechanistic analysis, particularly in terms of disassembly and escape kinetics. The suitability of this technique for the application of mRNA-LNPs requires the components to be labelled with a FRET pair dye, either mRNA/lipids or mRNA/endosome. Fluorescence signals changes are then monitored allowing the observation of mRNA dynamic release from LNPs [58, 59]. For example, the authors have monitored mRNA release from LNP using Cy5 labelled mRNA and DiR lipophilic dye by detecting Cy5 fluorescence recovery [22], while *Sahay et al.* used two identical siRNA labelled with different fluorophores [60]. Methods based on the pH property of the endosomal compartments, such as pH-sensitive dye-based microscopy, have also been used to study endosomal escape [61]. 

Overall, the assessment of RNA-LNPs trafficking and functionality is critical for understanding LNP properties. The major challenge is to determine the right in vitro model enabling a good prediction of their functionality. Unfortunately, in vitro assays are generally not harmonized between laboratories. Moreover, the poor correlation between in vitro assays and in vivo results remains a hurdle.

**Table 1:** 2D monoculture monolayer based assays used for the study of RNA-LNPs potency and trafficking

Test	Cell type	Organism	Tissue: cell line	Method
Determination of protein expression in mRNA/mRNA-LNP transfected cells	epithelial cells	Mouse	breast: 4T1 [58]	flow cytometry [58]
		Monkey	kidney: Vero [43]	fluorescence microscopy [43]
		Human	eye, retina: ARPE-19 [62] kidney: HEK293 [30–32, 37, 51, 62–69] liver: HeLa [33, 62, 63, 70, 71] - HepG2 [34, 72, 73] - Hep3B [73] - Huh7 [72] lung: A549 [63, 73, 74] ovary: SKOV-3 [73] - IGROV-1 [75] prostate: 22Rv1 [76]	western on cell lysates [64, 75] western on cell supernatant [64] flow cytometry [31, 51, 64, 66, 69–71, 73, 74] luciferase reporter assay on cell lysate [32, 63] luciferase reporter assay [30, 33, 34, 37, 64, 67–69, 72, 73, 75, 76] fluorescent microscopy [66] sandwich ELISA kit (GeneTex) [73]
	endothelial cells	Human	lung: HULEC-5a [36]	luciferase reporter assay [36]
	mesenchymal stem cells	Rat	rMSCs [37]	luciferase reporter assay [37]
		Human	hMSCs [37]	luciferase reporter assay [37]
	fibroblasts	Mouse	embryo: 3T3 [24] - NIH3T3 [36] large intestine, colon: CT-26 [65]	flow cytometry [24] luciferase reporter assay [36, 65]
		Hamster	kidney: BHK-21 [77]	flow cytometry [77] fluorescent microscopy [77]
		Human	lung: HEL 299 [62] skin: BJ [31]	western on cell lysate [62] flow cytometry [31]
	myoblasts	Mouse	muscle: C2C12 [37, 63]	luciferase reporter assay [37, 63]
	skeletal muscle cells	Human	skeletal muscle: HSkMC [24]	flow cytometry [24]
	dendritic cells	Mouse	DC2.4 [37, 51, 58, 67, 74, 77–79] - JAWsII (bone marrow) [77]- BMDCs [77]	flow cytometry [51, 58, 74, 77–79] luciferase reporter assay [37, 67]
	macrophages	Mouse	ascites: RAW264.7 [36, 37, 78]	luciferase reporter assay [36, 37] flow cytometry [78]
	T lymphoblasts	Mouse	B3Z T cell [78]	flow cytometry [78]
		Human	peripheral blood: JURKAT [36]	luciferase reporter assay [36]
mRNA-LNP trafficking (entry, endosomal disruption…)	epithelial cells	Mouse	breast: 4T1 [58]	confocal microscopy: uptake and intracellular trafficking of labelled mRNA [58] FRET: endosomal escape [58]
		Human	breast: MDA-MB-468 [80] kidney: HEK293 [81] liver: HeLa [24, 49, 56] ovary: SKOV-3 [82]	FRET [49] live cell imaging [49] High Content Imaging [80] flow cytometry [24, 82] confocal microscopy [24, 33, 49, 81] multicolor SMLM [56]
	myoblasts	Mouse	muscle: C2C12 [37]	confocal microscopy [37]
	dendritic cells	Mouse	DC2.4 [51, 58, 74, 79, 83, 84]	confocal microscopy [51, 58, 74, 79, 83, 84] FRET [58] live cell imaging microscopy [74]

### In vitro evaluation of RNA-LNPs toxicity

While the pharmacological and toxicological properties of nanomedicines are often investigated during in vivo preclinical studies, in vitro tests would enable optimal development of these therapeutics. In the field of mRNA-LNPs, cytotoxicity needs to be assessed for each component; mRNA [63] and lipidic components from LNPs [82] as well as the mRNA-LNPs complexes [51, 77–79]. Several in vitro tests, summarized in Table 2, are available to assess cytotoxicity and the induction of inflammatory cytokines’ release in specific cell lines. One of the most well documented cytotoxicity assays is MTT colorimetric assay (3-(4,5-dimethyl-2-thiazolyl)−2,5-diphenyl-2 H-tetrazolium bromide) based on mitochondrial activity [85–87]. For example DC2.4 cell line was well used for the evaluation of in vitro cytotoxicity of various LNPs [51, 79]. For inhaled formulations, pulmonary murine (MLE) or human (A549, Calu-3) epithelial cells commonly used for the evaluation of LNPs formulation [88, 89]. For instance, Calu-3 were used for the assessment of LNPs formulation cytotoxicity by performing real time impedance cell cytotoxicity study or MTT [90]. In addition, the lactate dehydrogenase (LDH) assay is broadly used for assessing nanoparticles toxicity by measuring the cell membrane damage caused by the release of LDH enzymes into the extracellular medium [86]. Using both MTT and LDH assays, researchers investigated the toxicity of mRNA-LNPs on HepG2 cells and highlighted that the MC3-based formulation induced higher cytotoxicity than the SM102-based formulations. There was no difference between the assays [91]. Nevertheless, some particles are known to induce some specific interactions such as LDH inactivation caused by metallic nanoparticles [92] or LDH adsorption [93]. 

Discontinuing preclinical development of nanomedicines is often related to nanoparticles reactogenicity inducing inflammatory reactions, complement activation, and disruption of the blood coagulation pathway [94]. The evaluation of inflammation through the study of activation pathways (e.g. interferon regulatory factor -IRF- and nuclear factor-κBeta -NF-κB- pathways) and pro-inflammatory cytokines release induced by mRNA-LNPs is critical, notably due to achieving an optimal product design with reduced adverse effects. mRNA vaccines may trigger innate immune responses LNPs, even in the absence of mRNA, activate immune cells, mediating the adjuvant role of LNPs and the release of proinflammatory cytokines (e.g. IL-1, IL-6, TNF-α) [21, 95–98]. In addition, the expression of IFN-β is highly specific to the mRNA component [63]. In this context, a wide range of in vitro immunology assays have been set up such as immunosorbent and multiplex assays to detect a variety of cytokines and chemokines released by the cells [99]. 

The assessment of mRNA-LNPs compatibility with human blood components is therefore fundamental to ensure mRNA-LNPs safety through administration. This is further exemplified through several cases of thrombosis reported in patients following mRNA-COVID vaccines administration [100]. In addition, other studies reported development or exacerbation of autoimmune hemolytic anemia in vaccinated patients, which is caused by the production of autoantibodies against red blood cells [101]. A risk evaluation in the early phase of development of mRNA-LNPs is essential to prevent life-threatening complications. Moreover, in vitro hematological assays such the study of hemolysis and platelet aggregation induced by RNA-LNPs could be assessed [102–104]. Finally, since complement activation can cause hypersensitivity reactions, thrombocytopenia or vascular inflammation, enzyme-linked immunosorbent assay or western blot are commonly used to study complement activation and thus mitigate clinical risks [105, 106]. 

**Table 2:** 2D monoculture monolayer based assays used for the study of RNA-LNPs cytotoxicity and pro-inflammatory cytokines

Test	Cell type	Organism	Cell line	Method
Cytotoxicity	epithelial cells	Human	lung: A549 [63] Calu-3 [90] liver: HeLa [63] – Hep G2 [91] ovary: SKOV-3 [82]	MTT [63, 82, 90, 91] LDH [91]
	fibroblasts	Hamster	kidney: BHK-21 [77]	cells viability by flow cytometry [77]
		Human	skin: BJ [63]	MTT [63]
	myoblasts	Mouse	C2C12 [63]	MTT [63]
	dendritic cells	Mouse	DC2.4 [51, 74, 77, 79] - JAWsII (bone marrow) [77] - BMDCs [77]	cells viability by flow cytometry [74, 77] MTT [51, 79]
	macrophages	Mouse	RAW264.7 [78]	AlamarBlue - fluorescence [78]
Measurement of pro-inflammatory cytokine production	epithelial cells	Human	lung: A549 [63]	ELISA: IFN-β, CCL5/RANTES [63]
	macrophages	Mouse	RAW 264.7 [107]	IL-6 [107]
		Human	THP 1 [108]	ELISA: IL-6 [108] multiplex: IL-1β, TNF-α, VEGF and MCP-1 [108]
	monocytes	Human	HMDM [108]	ELISA: IL-6 [108] multiplex: IL-1β, TNF-α, VEGF and MCP-1 [108]

### Limitations of 2D monoculture monolayer models

Monolayers models are limited by the lack of architectural complexity and biological heterogeneity [109]. These models are therefore poorly representative of the physiological response to a therapeutic drug. *Escalona-Rayo et al.* have shown that SM-102 based LNPs displayed higher in vitro protein expression and T-cell proliferation than ALC-0315 and Dlin-MC3-DMA LNPs. Meanwhile, in vivo studies in zebrafish and mice revealed almost identical protein expression levels for the three LNPs, without significant differences in antigen-specific immune responses [110]. In another study, the comparison between in vitro and in vivo nucleic acid delivered by hundreds of various LNPs to endothelial cells and macrophages concluded that in vivo delivery is not predicted by in vitro [111]. In contrast, in vivo animal models permit the evaluation of therapies in a more complex environment but still with obvious biological differences with the human organism. A major distinction between species is linked to the mass ratio between individual organs and the total weight of the animal, as well as the difference in immune responses, which are not conserved in all species. Indeed, biodistribution studies conducted in mice could reveal an overexpression of off-target liver delivery compared to humans [112]. LNPs delivery may also be impacted by metabolism variations between species. For example, *Havel et al.* investigated the importance of the use of non-human primates regarding metabolic diseases and concluded a greater similarity and susceptibility to metabolic disease with humans as compared to rodents [113]. Although these species are essential resources in pharmaceutical development for toxicity studies, their use is limited for ethical, practical and economic reasons, novel alternative methods that could provide relevant information are needed.

Overall, the low representativeness of 2D monocultured models has led to the development and emergence of alternative in vitro technologies in particular the 3D models with the benefit of traditional cell culture while respecting ethical debates. These 3D models offer a fitting microenvironment that closely resembles to the inner conditions of the human body, therefore generating more realistic responses.

## Toward a more predictive model

### Co-culture monolayer models

Although co-culture models were initially used for studying cell interactions, they are now widely used in drug development applications because they provide a more physiologically relevant model than 2D monoculture [114, 115]. 

Co-culture monolayer models can be divided into three categories including direct and indirect (transwell) models [116, 117]. Direct co-culture cells allow the cells to grow together in the same environment enabling physical contact and interactions, while indirect co-cultures use a physical barrier separating the cells but allowing the exchange of secreted factors. As the monocultures, the co-cultures involve either primary cells or immortalized cell lines. Several models are therefore being developed and largely described in literature, involving various cell types such fibroblasts, stem cells, immune cells as well as cancer cells, according to the studied disease [118]. For example, a direct co-culture model involving myoblasts from healthy human donors and dendritic cells (DCs) generated from human peripheral blood was used for the study of Idiopathic Inflammatory Myopathies, in the absence of in vivo models. The authors demonstrated that myoblasts and activated DCs interact closely during inflammation, inducing myoblast differentiation, and thereby increasing antigen presentation [119]. Another group reported that co-culturing of C2C12 muscle cells with RAW macrophages enhances inflammatory IL-6 secretion of the cells together, indicating a close interaction between skeletal muscle cells and macrophages [120]. These models could be perfectly extended to the study of the interaction of muscle cells and antigen presenting cells in the context of mRNA-LNP vaccination. In the field of lipid nanoparticles, *Yang et al*. studied the mechanism of internalization of anti-inflammatory nanoliposomes in human colon adenocarcinoma cells (Caco-2) and RAW 264.7 co-culture model, which mimics an intestinal inflammation system [121]. In their work, *Han et al*. engineered a co-culture platform for the screening of mRNA-LNP for the blood brain barrier (BBB). This model uses therefore endothelial cells and neuronal cells co-cultured in transwell, allowing the study of LNP transport and mRNA transfection through the system [122]. 

The advantages of the use of primary cells were described in the above section. BMDCs and splenocytes co-cultures are extensively used to study the mediated T cell immune response of mRNA-LNPs. In the context of cancer vaccine development, ex vivo matured BMDCs with mRNA-OVA model encapsulated in various LNPs were co-cultured with OT-1 CD8^+^ T cells presenting transgenic OVA-TCR [123–125]. The tolerogenic effect of mRNA-LNPs loaded with an anti-inflammatory drug was assessed in co-cultures of BMDCs and splenic CD4 + T cells, allowing the generation of antigen-specific T regulatory response for the treatment of various diseases such as allergies [126]. *Zhai et al*. mimic in vitro model for intramuscular vaccination by using transwell or direct co-culturing of human skeletal muscle cells and monocytes from healthy donors [127]. They observed that direct cell-cell co-culture enhances eGFP expression encoded by mRNA-LNP, increases antigen presenting cells (APCs) markers and the release of pro-inflammatory cytokines, particularly IL-6 playing a key role in B-cell activation [128]. These data highlight the importance of the stromal environment in the response to RNA vaccination.

### 3D in vitro models

The first in vitro cellular 3D models emerged in the beginning of the 20th century with Harrissons’s hanging drop technique and Leighton’s work on 3D cell culture using a cellulose sponge saturated with embryonic bird plasma [129, 130]. Scientific advancements in the following decades have led to the emergence of Complex In Vitro Models (CIVMs) including spheroids, matrix-embedded culture, organoids, 3D bioprinting and organ-on-chip (OOC) [131–135]. 

These technologies represent promising tools for more rigorous and representative studies of the efficacy and toxicity of therapeutic products. Most CIVMs involve a co-culture environment meaning that two or more different cell populations are grown in vitro together, with varying degrees of contact or a shared environment. In that way, these models produce better tissue architecture, cellular interactions, and microenvironment conditions than traditional 2D monocultures, bridging the gap between conventional 2D in vitro tests and animal models [109, 131, 136]. 

**Fig. 1:**
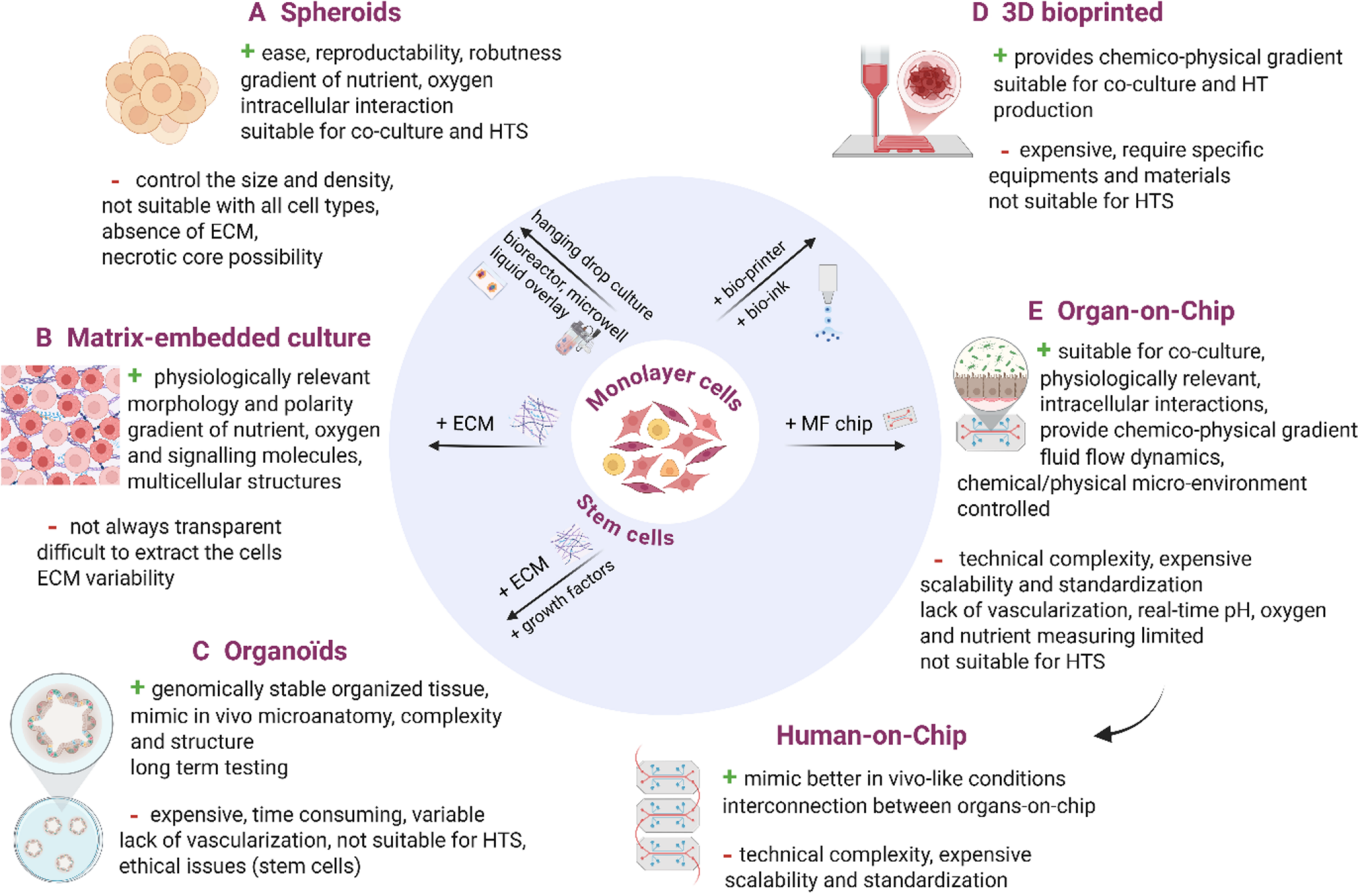
Schematic representation of multilayers in vitro models. Main 3D models, their advantages and limitations are presented

Studying the key effects of RNA-LNPs on CIVMs would be more relevant and predictive as the cellular functions are highly dependent on 3D organization [137]. A variety of CIVMs have been developed, differing by their technical complexity and tissue organization (Fig. 1).

#### Spheroids

**The spheroid** model (Fig. 1.A) is the simplest 3D culture model currently used. It is defined as spherical aggregates of cells which mimic spatial architecture and promote the recovery of original tissue morphology and functions. Several groups have studied the toxicity of nanoparticles (NPs) on 3D cellular assays, notably using spheroids, and have compared the results with 2D monocultures and in vivo results [138–140]. 

In other studies, spheroids have been used for the evaluation of mRNA-LNPs trafficking and the determination of protein expression after being treated with fluorescent-labelled particles and fluorescent-labelled mRNA [141–144]. *Kim et al.* have, for example, investigated the penetration within L929 spheroids of PE-rhodamine-labelled LNPs with prazole adjuvant (ESO-LNPs) and Cy5-labelled eGFP mRNA, highlighting their enhanced penetration compared to traditional LNPs (without adjuvant). The eGFP protein expression had also been determined using confocal microscopy, revealing an improved expression using ESO-LNPs. Using Western blot, they also demonstrated the ability of these formulations to inhibit the extracellular matrix (ECM) signaling pathway leading to an improved penetration of LNPs and mRNA [143]. Even though spheroids are a straightforward, reproductive and robust predictive model, it is important to consider some limitations related to technical aspects such as controlling spheroid size and cellular density.

#### Matrix embedded culture

**Matrix-embedded culture** (Fig. 1.B) is a model where matrix components (e.g. collagen, fibronectin.) are added into cell culture to mimic the ECM. Thus, the cells grow in all directions with more appropriate mechanical, biochemical, and spatial cues. This model furnishes a closer in vivo environment and physiological cell behavior, rendering it a promising tool for drug testing [145–147]. *Belly et al.* have notably investigated the mechanism of polystyrene nanoparticles uptake on 2D monolayer cultures and 3D collagen-matrix-embedded cultures. They have demonstrated that the endocytosis process is enhanced in 3D arrangements thanks to the ECM and the 3D structure, which increases the amount of cell surface available for NPs internalization. To the best of our knowledge, the potency and safety of LNPs were not assessed on matrix embedded culture, the main authors using more complex models like organoids.

#### Organoids

**Organoids** (Fig. 1.C) are 3D structures derived from stem cells (pluripotent stem cells, neonatal or adult tissue cells, adult progenitors), which differentiate and self-organize spontaneously [131, 133, 148]. Despite the complexity of organoids development, they are among the most relevant CIVM, as they closely resemble in vivo characteristics. In the field of nanoparticles, induced pluripotent stem cells (iPSC) - derived hepatocyte cultures have been used to evaluate mRNA-LNPs uptake using a luminescence counter and to quantify protein levels in the culture medium after transfection using ELISA [149]. *Bayraktutan et al. *evaluated the effectiveness of polysarcosine based polyplexes in delivering self-amplifying RNA (saRNA) to intestinal organoids, which naturally mimic some aspects of real organ physiology. Using confocal microscopy, they demonstrated the high expression of GFP within the dense matrix of cells in the deep core of the organoid, indicating nanoparticle penetration [150]. Using patients’ biopsies, researchers have optimized episkin organoid by the addition of PBMCs isolated from patients to investigate microRNA-lipoplexes in the context of cutaneous lupus erythematosus (CLE). They demonstrated that these lipoplexes induce effective cellular penetration, low cytotoxicity and successfully modulated inflammatory pathways in the immune 3D skin organoid [151]. 

#### 3D bioprinted model

**3D bioprinting** (Fig. 1.D) is one of the most automated and controlled methods, as it involves layer-by-layer deposition of biological materials such as cells or bio-inks (e.g. cells, growth factors.) from a pre-defined digital design model. This process mimics the architecture and function of natural tissues, producing tissue-like constructions. Similarly to other CIVMs, the 3D bioprinting model is used to study diseases and to develop new drugs [152–154]. A 3D bioprinted model could therefore be useful for guiding the development of NPs prior to animal studies. For example, researchers have developed a reliable tumor model that mimics biological characteristics, including the ECM barrier, gradients, and cellular interactions. Using this model, they evaluated cellular uptake efficiency and the effect of ECM barrier on the penetration of the nanoparticles in the tumors [155]. *Zhuang et al.* reviewed various applications and methods used to form muscle fibers [156]. For example, *Gholobova et al. *evaluated the impact of multiple intramuscular injections and the release of the injected compound using human muscle tissue biopsies [157]. Some authors also incorporate immune cells or endothelial cells into the model to study vaccine behavior [158, 159]. In another way, an example of a multilayer model is the 3D dermo-epidermal skin (DESE) constructed by *Akagi et al.*, which was evaluated as an alternative to skin permeation and irritation tests [160]. This approach could be used to study vaccines and to track the fate and toxicity of RNA-LNPs after injection. For the best of our knowledge, literature has not reported studies evaluating RNA-LNPs in 3D bioprinted models. Otherwise, immunocompetent skins are currently available from companies such as Genoskin^®^ for the testing of drug efficacy and safety which could be applied in RNA-LNP development.

#### Organ-on-chip

**Organ-on-chip** (Fig. 1.E) mimics the fluid environment of living cells native to organs, reproducing on a small scale their key characteristics such as cellular architecture and functionality. This model offers also a tuned dynamic environment thanks to the high control over liquid flow in microfluidic chips. Today, a wide range of organs-on-chips have been successfully developed to investigate drug potency and toxicity [161–163]. Lymphoid organoids and organ-on-chip methods using PBMCs and tonsil cells have been employed for vaccines testing previously [164]. *Goyal et al*. developed a human lymphoid follicle (LF) showing many features of germinal centers found in lymphoid organs using primary human cells from PBMCs. They demonstrated that human LF Chips can be used to evaluate the vaccine immune responses in vitro [165]. In the field of mRNA-LNPs, *Jeger-Madiot et al.* used the same system to model the memory B-cell response to COVID19 mRNA vaccine. They were able to study B lymphocytes proliferation and antibody production after prime and boost administration of mRNA-LNPs [166]. Recently, the same group introduce a biomimetic module containing human skeletal myoblasts and antigen-presenting cells (APCs) to mimic intramuscular vaccination, followed by transfer of the activated APCs and soluble factors to the LF Chip to mimic lymphatic drainage. This offers a powerful alternative for evaluating mRNA-vaccine induced immunity in a relevant human model [127]. 

## Toward in vivo replacement models

In the context of the 3Rs, the relative replacement of animals with less sensitive ones is recommended. The **chorioallantoic membrane** (CAM) assay is based on the strongly vascularized membrane found in fertilized chicken eggs. It is therefore used for a panel of testing such the study of vascularization, damage and irritation, in vivo circulation and finally the determination of median lethal dose (LD50) [167, 168]. Furthermore, the CAM assay is known as an alternative option to the Draize test allowing evaluation of potential irritation of topical formulations, earlier in the development, before animal studies [169]. The biocompatibility of LNPs developed for eye-controlled delivery of anti-inflammatory or antioxidant drugs were evaluated by the CAM assay. *Almeida et al.* demonstrated the absence of in vitro and in ovo cytotoxicity of their LNPs evaluated by the Alamar Blue assay using the Y-79 human retinoblastoma cell line and CAM assay after exposure to increasing concentrations [170]. Interestingly, F*anguiero et al.* observed a similar non-irritant profile of their cationic LNPs tested in the CAM assay and evaluated by the Draize-test in rabbits [171]. In another context, the potential irritation of unloaded LNPs and photosensitizer-loaded LNPs were assessed by CAM, before being used for tumor treatment. No irritation such as hemorrhage, lysis, and coagulation occurred, confirming the biocompatibility of these LNPs [172]. The development of safe and localized skin delivery system to improve drug availability and avoid systemic toxicity was also investigated. *Passos et al.* developed nanostructure LNPs delivering an antifungal drug for the treatment of fungal skin infections. They demonstrated, using the CAM assay, that unloaded and drug-loaded nanocarriers induced very low irritation scores, enabling their use in skin lesions without generating skin damage [173]. 

For biomedical research, the **zebrafish** (*Danio rerio*) is one of the most used alternative models. It offers a combination of practical, biological and genetic advantages, making it an ideal model organism for studying human diseases, developing new drugs and nanomaterials. In fact, they display a significant degree of hereditary resemblance to humans, around 70%, as well as a high fertility rate (approximately 300 eggs produced by a single female). In addition, the transparency of the embryos and their rapid development make it possible to perform many tests easily to investigate cellular interactions, organ-specific accumulation and circulation time [174–177]. Finally, the rapid development of embryos allows the study of drug effect on several zebrafish generations.

In recent years, zebrafish has been proposed for the assessment of potency and toxicity of mRNA-LNPs. In their review, *Bondue et al.* illustrate how zebrafish can be used for cost-effective and high-throughput in vivo testing of mRNA-LNPs in genetic disease models (e.g. Duchenne muscular dystrophy, Classic galactosemia.) before testing in rodents [178]. Within the field of veterinary research, a recent study demonstrated the first proof-of-concept for the efficacy of RNA vaccination in fish. It was therefore shown that LNPs-based mRNA vaccine candidate encoding the hemorrhagic septicemia virus (VHSV) induce neutralizing antibodies, therefore offering a complete protection in zebrafish [179]. 

In addition, the biodistribution of mRNA-LNPs could be assessed in the zebrafish model. For example, *Patton et al.* reported that following injection, GFP mRNA-LNPs were delivered to multiple tissues without any toxic effects on embryonic development. Subsequently, the GFP protein was expressed in several tissues such neural, vascular, cardiac, and skeletal muscle tissue, depending on the administration route [180]. In another study, zebrafish was used for the rational design of LNPs targeting the reticuloendothelial system in the liver, avoiding the conventional unnecessary screening in animals. They observed concordance in LNP uptake pathways in zebrafish and rodents, rendering zebrafish a powerful model for LNPs discovery [181]. 

To improve the safety of mRNA-LNP therapeutics, research groups focused on the optimization of either mRNA sequences or LNP composition. Indeed, it has been reported that PEGylated LNPs induced hypersensitivity reactions and accelerated blood clearance (ABC). This phenomenon is likely due to the presence of pre-existing anti-PEG antibodies in patients [182]. For this purpose, *Bi et al.* developed a PEG-free formulation by the substitution of PEGylated lipid by polysacrosine (pSar) lipopolymer. They showed the efficacy of pSar formulations to deliver mRNA either by intravenous route or intracranial route in the central nervous system of zebrafish without any acute toxicity [183]. Furthermore, high doses of mRNA COVID19 vaccines induced adverse events in the patients. To diminish mRNA reactogenicity while increasing its stability and potency, *Fernandez et al.* have shown that optimization of untranslated regions, as well as purification methods, increase the potency of naked mRNA in zebrafish after intramuscular administration [184]. However, validation in rodents and bigger animals is suggested by the authors for better predictivity.

## In silico models for the prediction of potency and toxicity of RNA-LNPs

The phenomenal progress in computational sciences in the last decades allowed and accompanied the development of a wide variety of bioinformatics tools which, recently, raised interest in research related to RNA-based therapies. In addition, the increased use of automated processes and high-throughput methods allowed to generate large amounts of data, which is essential for the development of pharmaceutical products. The use of bioinformatics and artificial intelligence (AI) based tools could provide information and relationships with reduced resources and time.

The bioinformatics tools sustained by experimental data from various sciences, give access to upstream optimization of therapeutics, such as biologics, immunomodulators, vaccines, vectors and their components. These optimizations may concern an extremely vast range of characteristics of these therapeutics. For instance, it allows (i) to provide information regarding their interactions with cells, as T-cell epitopes, regulatory T-cell epitopes, patterns of self-T-cell epitopes, (ii) to attempt predictions of nucleic acids, proteins and small molecules properties and their interactions, as tridimensional protein structures, the protein-protein complexes formation, intermolecular interactions, protein and nucleic acids secondary structures and (iii) to predicate physico-chemical properties such as solubilities, hydrophobicities, pKas, isoelectrical points, apparent sizes, etc [185–192]. 

In addition to these benefits, bioinformatics tools allow to obtain optimized products and strategies towards related advantages such as the stability of sequences, secondary structures or even lipid vectors in the case of mRNA vaccines for example. The implementation of these new abilities will directly impact the effectiveness of the drugs and thus, allow the reduction of doses to be injected and consequently the potential deleterious effects of treatments [188]. 

Bioinformatics tools also enable to optimize personalized medicine development. For example, the analyses of individualized T-cell epitopes or mutanomes allow the design of personalized therapeutics in the context of oncological treatment [185]. These tools could also be used to achieve an optimal therapeutics outcomes establishing nonlinear relationships between drug candidates and their potency and toxicity.

Applying computational tools and in silico models in the early stages of therapeutics design, antigen selection, and engineering is likely to result in the advancement of next generation products with minimum off-target or non-expected deleterious therapeutics efficacy [185]. 

The use of in silico models is hindered by some limits. As an example of a key limitation of bioinformatic modeling, protein structure prediction models typically predict static structures, and not the dynamic behavior of biomolecular systems in solution [186]. Furthermore, the progress of bioinformatics tools, and even more those relying on the latest statistical learning methods, such as machine learning, based on neural networks or deep learning, depend necessarily on the support of a large database of in vitro/ex vivo or in vivo experiments [185, 186, 188, 189]. The availability of a large set of high-quality data, that produced under standardized and validated protocols would unlock the full potential of machine learning (ML). Recently, several studies reported the application of machine learning tools in the field of nanomedicine [193–196]. ML allowed, for example, the prediction of the cellular internalization of PLGA NPs [80] and the biodistribution of gold NPs [197] using a set of experimental data produced in-house. This approach could be applied to predict and optimize the in vivo performance of mRNA-LNPs. In this context, the use of AI for the screening of new ionizable lipids was reported and allowed the prediction of the apparent pKa of LNPs, which is closely related to the efficiency of mRNA delivery [198]. Recently, a new study described the use of ML to analyze a data set of LNPs formulations and to find relationships that linked the lipid composition and chemistry to transfection outcomes [199]. 

The effective integration of in silico immunoinformatic tools, ex vivo/in vitro and in vivo immune system technologies, and ML tools across the entire therapeutics development pipeline will allow to predict and assess safety, toxicity, efficacy, quality, and performance of drug candidates. This will certainly accelerate the development of safe, and effective immunotherapy for a broad range of pathogens and species.

## Conclusion

mRNA-based therapeutics represent a transformative advancement in biomedical science and gene delivery. The emergence of this new class of drugs has been accompanied by confusion concerning the analytical procedures for the quality analysis of mRNA and mRNA-LNPs, due to a lack of guidance and inconsistency between regulatory agencies worldwide. While physico-chemical characterizations are quite well established, recommendations for in vitro potency and toxicity testing are still far from being comprehensive.

In this review, we provide a comprehensive and analytical overview of the in vitro toxicity and potency evaluation of mRNA-LNPs. The strengths and limitations of the currently used models and protocols, as 2D monolayer monocultured models, were explored. It is important to bear in mind the limitations of these models, particularly regarding their lack of representativeness of in vivo study results.

Currently, animal studies are still required by regulatory authorities, but the lack of representativeness of animal models and low predictivity for human have led to the development of new alternatives methods. Therefore, we discussed co-cultured 2D models and progressing to multilayer models such as spheroids, organoids, and organ-on-chip models. The key benefit of these models is that they enable the microenvironment and the architecture of the tissue to be replicated. Today, spheroids and organoids are well established in the research development of metallic and solid nanoparticles allowing their future use in the RNA-LNPs field. Moreover, the most cutting-edge CIVM is based on the parallelization of several organ-on-chip systems to approximate human physiology as closely as possible. These systems are generally referred to “human-on-chip” or “body-on-chip” which are used to predict the pharmacokinetics and pharmacodynamics of therapeutic products [106, 200–203]. Nevertheless, 3D multi-layer models continue to be costly and technically more challenging to develop and scale-up. In addition, these models are not widely used in the field of RNA-LNPs yet, most likely due to the limitations highlighted in this review. Other alternative models such as CAM and zebrafish are increasingly described in the literature for the study of RNA-LNPs functionality and toxicity. They offer a more complete environment and request fewer operating conditions. Moreover, they represent a real advantage for high-throughput screening of new LNPs due to their low cost. On the top, computational methods for the optimization of RNAs and LNPs could be introduced at all stages of the workflow, allowing a better selection of the formulation and reducing time and cost of development. Overall, this synthesis allowed us to highlight the landscape of available tools to guide the selection of most relevant models, ensuring the optimal preclinical evaluation. 

In a nutshell, there is no consensus on how best to assess the potency and toxicity of RNA-LNPs in vitro. Although, the accelerated development of novel alternative methods (NAMs) could bridge the gap between in vitro studies and animal research, allowing more human predictive data and more specifically for sub-population groups who are not eligible for clinical trials (e.g. women pregnant, children.). Discoveries in lipid and mRNA chemistry and biology, advances in computational sciences, the use of high-throughput methodologies for screening, and the development of innovative models with high relevance will be vital to translate mRNA technology into clinically viable solutions.

A collaborative strategy that engages making decision stakeholders to establish clear, harmonized and standards criteria for the evaluation and validation of NAMs will accelerate their regulatory acceptance, benefiting human health with respect to 3Rs. In this context, plans and measures (e.g. increased resources for the development of new alternative models, the implementation of educational and training programs on the 3Rs) have been introduced by the European Commission and the FDA to accelerate the transition to animal-free innovation in research.

## Data Availability

Not applicable.
